# Some Practical Points Learned at Society Meetings and Clinics

**Published:** 1890-05

**Authors:** B. A. R. Ottolengui

**Affiliations:** New York City


### SOME PRACTICAL POINTS LEARNED AT SOCIETY MEETINGS AND CLINICS.[1]

BY DR. B. A. R. OTTOLENGUI, NEW YORK CITY.

EXPERIENCE is the best teacher. However thorough the college training may be, or become, no man will ever graduate with as much practical knowledge as will come to him by actual work at the chair. If experience, therefore, is a great teacher, and each man is taught by different experiences, it must be a fact that different men learn different methods of accomplishing the same thing, and also learn to do different things. What then can be better than meetings and clinics where these differing methods may be exhibited and discussed? Suppose we read on a programme, "Dr. A—— will fill a tooth with gold;" must we say, "We can learn

---

[1] Read before the Brooklyn Dental Society, December 25, 1889.

nothing there, we all know how to fill teeth with gold?" That would be a great error. We may learn nothing, but then we may learn something, and that possibility makes it to our advantage, if not our duty, to attend that clinic. It is almost impossible to go into the worst-appointed dental office without seeing something new, or at least different from our own methods. We are all students, and at the same time we are all teachers. We should be willing in both parts.

To-night I have to offer you a brief but practical paper. I think nothing in it is entirely original with myself. I suppose that there is not any idea in it which some of you have not heard before, but I doubt if there is any one who can say, "I knew them all;" and if each man gets but one new idea to-night he will be repaid. These little methods have been extremely valuable to me, and I could not dispense with any one of them.

I will begin with the rubber dam. Probably no dentist will admit that he is not master of so simple a thing as the rubber dam; and yet how often does the dam become the master of the dentist merely because some unforeseen accident occurs in the midst of an operation. There is the tiniest tear through which mucous will ooze; the dam did not pass entirely down between the teeth, and moisture is creeping towards our work; the clamp slips; we have not allowed quite enough margin to the rubber to cover the mouth; we thought we had, but when we applied the clamp we discovered our error, and so on *ad infinitum;* through some little oversight we have failed in that seemingly simple operation the application of the dam. So much annoyance has occurred in this manner that perhaps you will pardon me if I think it important enough to tell you all the little tricks which I have learned in this connection.

For comfortable work, the rubber, when in position, should embrace at least four teeth; on dark days it is not amiss to take in twice as many. It should lay over the face without a wrinkle, and should not cover the nostrils; it should, however, completely cover a moustache, as the hairs often intervene between our eyes and work. To accomplish this a piece of dam of sufficient size should be stretched over the parts which it is intended to cover, so that the proper position for the holes may be ascertained, allowance being made for the stretching which will be made by the clamp. In this position the cusps of the teeth will show through the rubber, and a mark over each may be best made with an excavator, a pencil not answering as well. If because of the loss of a tooth a space must be spanned, the rubber should not be stretched at that point; if this is not considered it will be found that when the dam is stretched over the teeth it will not hug the necks of the teeth at this point. In fact, this rule holds for all spaces great or small; the rubber should be wide enough. In cutting the holes use a device which makes a perfectly round hole, this being the least likely to tear. Make the holes sufficiently large; don't force a molar through a hole which would be just right for a bicuspid. Where the teeth are in close contact, soap a bit of waxed floss silk and pass it between all the teeth first; then soap the edges of the holes in the dam; in this manner there is seldom any difficulty about forcing the rubber between the teeth. Occasionally, even this will not serve. Your predecessor (of course not yourself) has left a filling with ragged edges, which tear the rubber. In this case the teeth in question should be wedged with soaped wood, as will be described later. The least spreading allows the rubber to pass between, when the wedges may be removed. This is better than trying to force the dam between the teeth with silk. That method not unfrequently tears the rubber, and accounts for the mysterious oozing which occurs whilst the filling is in progress, and is largely responsible for the failure so often reported at the cervical border. If the dam has been properly adjusted, it can be removed in perfect condition. How often have you noticed, after removal, that in addition to the holes made by your punch there are several others, satellites, as it were, about the greater orbs.

Next comes the clamp. In the first place select the one to be used before applying the dam. Choose one which will grip the tooth tightly. Throw away all clamps which would not hurt you if put on your finger. A clamp without a spring is no better than a clock in the same condition. In applying the clamp to a molar in the upper jaw a little trick is found to be most valuable. We begin by slipping the rubber over a central incisor, then over the lateral cuspid and bicuspid, and finally over the first molar, let us say. We endeavor to apply the clamp and find little room, and the patient flinches. The cause is this: The middle finger is the one we use to adjust the dam; it protrudes into the mouth, and as we work towards the molar region we gradually fold the angle of the mouth inward so that at last it is held back by the tip of the finger, and it is difficult to find room for the clamp. Just at this point, take the handle of a burnisher or other instrument and free the check so that the finger passes into the mouth, the check slipping forward; then it will be found that, not being crowded back, its elasticity gives us sufficient room to apply the clamp without pain. This one point has been of inestimable value to me, and to my patients in saving pain.

Before passing to ligatures there is a special case to be alluded to. Where the gum has receded and a large festooned cavity is present, the space on either side of the hole which is to embrace the tooth to be filled should be wider than ordinarily made; otherwise, when stretched so far up on the gum, there will be leaking about the edges. Ligatures should be dispensed with as much as possible. They are frequently the cause of more pain than any other part of an operation. It is rarely necessary to ligate more than two teeth, and frequently no ligature at all is needed. The trick is done by inverting the edge of the rubber so that it slips under the margin of the gum; if the root is at all conical, the elasticity will cause the rubber to crawl up and tuck itself under nicely. If a ligature must be used, a little cocaine is useful. There will come to us cases where the ligature is absolutely necessary, and where it seems almost impossible to place it so that it will not ride up around the crown rather than remain at the gum margin. Let us suppose such a case in connection with an upper lateral incisor. The cavity is in the palatal sulcus, therefore the ligature must be forced up. The trick is to tie a good knot in your silk first; placed about the tooth, this knot must come at the centre on the palatal side; it makes a good point of resistance for the instrument, and is pressed up under the margin of the gum, carrying the rubber with it; the gum contracting holds it, and when tightly tied on the labial side holds securely. This is the first point I ever picked up at a clinic, and, as I have never seen it at one since, I would have lost a great deal of satisfaction which it has brought me had I been absent from that clinic.

I alluded to leaking. In a very wet mouth, after the best precautions, ligatures well placed, it will sometimes happen that moisture will creep in around the neck of the tooth. Take a piece of spunk, dip it in gum sandarach, being careful not to get an excess, and pack it in a rope around the neck of the offender. Then apply a second ligature which shall tie the spunk in place. The leak will be stopped. If an instrument has slipped and torn a small hole, it may be stopped with a bit of sponge dipped in sandarach. Where the leak is about a clamp, the clamp should be taken off carefully, a fairly large piece of spunk, treated as described, placed along the edge of the rubber, and the clamp reapplied so that it bites the middle of the spunk holding it in place. As to the slipping of a clamp, it sometimes occurs because the dam is held too tight by the rubber strap which passes around the head, or there is a strain from the dam weights.

In some cases it will be found impossible to apply the dam at all. There is a way of using the napkin which may not have occurred to all. A small mouth napkin is rolled into a narrow fold, and placed about the tooth in the shape of the letter "U," the ends forward. It is so arranged that the folds extend slightly upon the sides of the tooth where it is firmly held in place with a clamp. There is a special clamp made for this purpose by Dr. Ivory, but any clamp of suitable form will answer.

There are a few points about oxyphosphate fillings worthy of note. We have all noticed that what is left on the mixing dish is usually more adherent and harder than what we put into a cavity. Both these facts depend on circumstances which are usually absent in the mouth. To make a dense filling it should be allowed to set thoroughly before the dam is removed, and moisture should be excluded for at least twenty-four hours. This may be accomplished by using a coating of chlora-percha over the finished surface of the filling. If the dam is left on until this varnish has hardened by the evaporation of the chloroform it will not wear off for a week, and I have known it to last two months. Such fillings are comparatively permanent. Where we wish to utilize the sticking or cement quality of this material, the best result is obtained by first lightly coating the surfaces with the liquid. This is why the material is so adherent to the slab. I have thus cemented regulating fixtures to teeth, and at the completion of the work found it troublesome to detach the cement from the enamel after the fixture had been forced off.

In teeth which are sensitive, and where a good general shape to the cavity is present, do not make a retaining pit or groove for starting the filling. I am aware that some of you will say, Never do so under any circumstances. But possibly you preach better than you practise, and you may make such a pit or groove to-morrow if you find it more convenient. In sensitive teeth, whether the pulp be nearly approached or not, a coating of oxyphosphate between the filling and tooth is an advantage; place it there and then press gently two or three pellets of gold into it, without attempting to condense them. When the cement has set, carefully chip away such portions as have crept over the edges, and the gold thus cemented to the tooth forms the very best starting-point. Of course amalgam may be used similarly.

Gilbert's temporary stopping is furnished us in two colors, red and white. At the first glance it would seem that the white is to be used in the front of the mouth and the red out of sight. The difference in color can be better utilized than that. Use the red to cover arsenic dressings or in any place where immediate continuance of treatment at the next sitting is imperative. Use the white for teeth in a comparatively safe condition. In this manner, as soon as the mouth is examined, the observance of the red filling is as a danger signal, and calls our attention to the fact that there is something which may not be postponed.

A gentleman said at one of our meetings recently, "I have several kinds of separators; I could not get along with only one." I made a mental note at the time that this was odd, because I get along without any. I think the separator is a dangerous instrument. It is in rare cases only, where good and sufficient space may be thus acquired, and in unskilful hands, especially young practitioners, the probability of failure at the cervical border is, in my opinion, increased tenfold. If a tooth is to be filled, the first and most important point is that the completed filling shall be perfect. There are few men who can put in as good a filling in a space barely admitting an instrument, and there are fewer still conscientious enough to do it, even granting them the skill. The best teaching then for the young, and I think for the old as well, is to depend on the rubber or wooden wedge. There is a trick in the application of each. When using rubber allow a bit of it to protrude below the cutting ends of the teeth. This part, by contraction as the teeth move, will swell, and the rubber is prevented from pressing up against the gum. To apply the wooden wedge, proceed thus: The wedge is trimmed to the proper width and should approach a taper very gradually. If it is then made smooth with a bit of sand-paper it will be less likely to split. Lastly, it should be soaped. A second wedge should be made quite thin, and have a shoulder which will prevent it from passing between the teeth beyond that point. This, also soaped, is placed between the teeth next to the gum temporarily. Now, when the permanent wedge is forced into position, this one first placed prevents it from hurting the gum and offers a slippery surface for it to slide against. The wedge in place and trimmed to suit, the temporary slip is removed, and this relief of pressure against the gum is gratefully acknowledged by the patient. At the next sitting, supposing the teeth separated but quite sore, gutta-percha should be placed between them and worn for several days. There is a neat trick about this. If the material is softened it is frequently difficult to fix it tightly in place. Cut a piece from a sheet and press it into place cold, then smooth and trim into shape with warm burnishers.

Occasionally, in soldering, a portion of our investment breaks off, exposing a part of a tooth. We can ill afford the time to patch the break and wait for the plaster to harden again. The exposed portion of the porcelain may be perfectly protected by covering it with a thick paste of chalk and water. This mixture may also be used to fasten small pieces of gold to the solder-block while soldering.

You all have seen artificial dentures where, after brief wearing, the front blocks separate, the plate finally breaking in half. To avoid this, permanently unite the blocks by soldering a platinum bar to the pins. To do this, after the teeth are ground to proper position, make a guide with plaster along the outer surfaces and then take off the front blocks. They are set into position in this guide, waxed together and invested, when the soldering may be done, the blocks dropping back into proper place.

There is nothing better than the pins from old teeth, soldered to a gold plate, for securing rubber attachments. The graving of a plate or even punching holes is a delusion and a snare. The rubber will separate from the plate some day. The prettiest and strongest plate is made with what we know as "celluloid" teeth, soldered to a gold plate and then rubber vulcanized around them. The plate teeth are not made in as good moulds.

Don't varnish plaster impressions. Soap the surfaces with a shaving brush. Be careful to wash off the suds, or the model will be pitted. Put a little red paint in the water when pouring your model, and in separating, the model is easily detected, by its color, from the impression.

Occasionally a gold plate is brought to us with a tooth broken off, the pins of course remaining in the backing. It may be that a good match cannot be found, or you may be in a hurry, so that you wish the same tooth could be used. Proceed as follows: Boil the tooth in acid to get the stump of the pins remaining as clean as possible. Invest it as for a backing. Lay a bit of pure gold over each broken pin and point a fine flame with the blow-pipe till a tiny gold ball is made on each broken pin. These may be filed up and will be sufficiently long to allow backing the teeth, using platinum foil and gold of a lower carat.

